# Clinical Remission and Reduction of Circulating Nephritic Factors by Combining Rituximab With Belimumab in a Case of Complement Factor 3 Glomerulopathy

**DOI:** 10.1016/j.ekir.2024.02.1402

**Published:** 2024-02-24

**Authors:** Mieke van Schaik, Aiko P.J. de Vries, Frederike J. Bemelman, Ton J. Rabelink, Leendert A. Trouw, Cees van Kooten, Yoe Kie Onno Teng

**Affiliations:** 1 Center of Expertise for Lupus, Vasculitis and Complement-mediated Systemic disease (LuVaCs), Department of Nephrology, Leiden University Medical Center, Leiden, The Netherlands; 2 Department of Nephrology and Leiden Transplant Center, Leiden University Medical Center, Leiden, The Netherlands; 3 Department of Nephrology, Amsterdam University Medical Center, Amsterdam, The Netherlands; 4 Department of Immunology, Leiden University Medical Center, Leiden, The Netherlands

## Introduction

Complement factor 3 glomerulopathy (C3G) is caused by dysregulation of the alternative complement pathway (AP), resulting in either dense deposit disease or complement factor 3 (C3) glomerulonephritis. Both diseases are characterized by a distinct pattern of glomerular C3 deposition in the absence of immunoglobulins, frequently producing a membranoproliferative glomerulonephritis pattern of injury.^1^, ^2^, ^3^ Patients commonly present with a nephrotic or nephritic syndrome and often with low serum C3 levels, and the diagnosis is confirmed with a kidney biopsy. In 50% to 80% of cases, C3 glomerulonephritis is associated with antibodies targeting various complement components.^4^, ^5^, ^6^, ^7^ Among these, C3 nephritic factors (C3NeFs), autoantibodies that stabilize the AP C3 convertase amplifying the complement cascade, are most common, occurring in up to 80% of patients with dense deposit disease and up to 50% in C3 glomerulonephritis.^4^^,^^6^ Data on the prevalence of complement factor 5 nephritic factors (C5NeFs), autoantibodies against complement factor 5 convertase, are less robust; 1 study identified C3NeFs in 50% of cases.^7^ Forty percent of patients have both C3NeF and C5NeF, and less than 10% of patients exhibit other autoantibodies, against the classical pathway C3 convertase (complement factor 4 nephritic factors [C4NeFs]), factor H, or factor B.^4^^,^^8^ Although the C3 convertase stabilizing capacity of C3NeF is associated with disease progression, their precise contribution to the pathogenesis remains unclear.^9^

There is no approved therapy for C3G. Treatment relies mostly on glucocorticoids and mycophenolate, based on limited, low-quality evidence from uncontrolled cohort studies.^6^^,^S1 No clinical advantage has been demonstrated by removal of autoantibodies through plasma exchange, and there is unconvincing success of B-cell depletion with rituximab.^4^^,^S2 Consequently, nearly half of the patients progress to end-stage kidney disease within a decade, and disease recurrence after kidney transplantation is common, with a 5-year graft failure rate of 50%.^3^^,^S1,S3,S4

Here, we present a patient with treatment-resistant C3NeF-positive and C5NeF-positive C3G with end-stage kidney disease, who achieved a remarkable response through the innovative approach of combined B-cell targeted treatment with rituximab and belimumab, thereby averting the necessity of kidney transplantation, and significantly reducing autoantibody levels.

## Case Presentation

**Figure 1:**
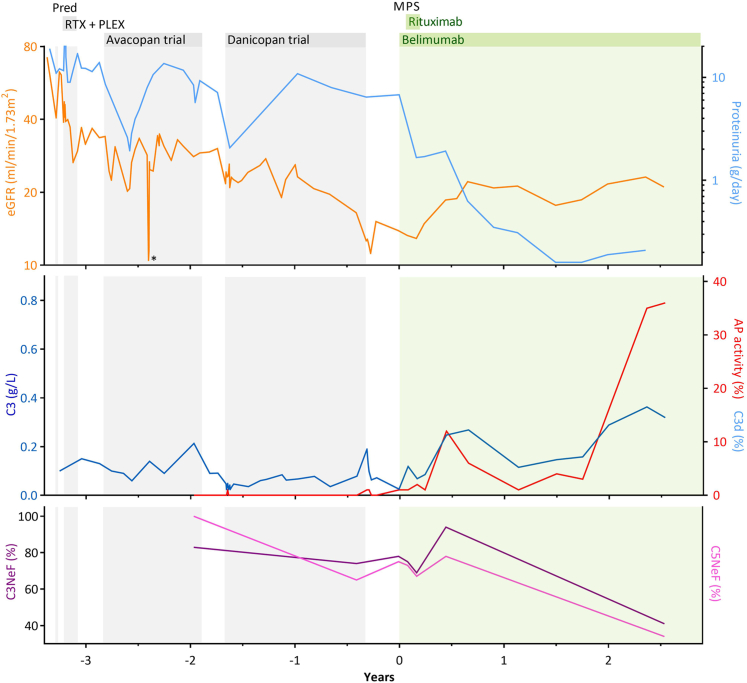
Treatment response. Course of eGFR and proteinuria (top panel) and complement biomarkers (middle and bottom panels) over time and through the different courses of treatment. ∗Temporary rise in serum creatinine due to an episode of infectious colitis. Reference values: eGFR, >90 ml/min per 1.73 m^2^; proteinuria, <0.15 g/d; C3, 0.9–2 g/l; C3d, 0.5–3.1%; AP activity, >39%; C3NeF, <20%; C5NeF, <20%. AP, alternative complement pathway; belimumab, 200 mg subcutaneous belimumab; C3, complement factor 3; C3NeF, C3 nephritic factor; C5NeF, complement factor 5 nephritic factor; eGFR, estimated glomerular filtration rate; MPS, 3x 1g i.v. methylprednisolone; NeF, nephritic factor; PLEX, 7x plasmapheresis; Pred, 80 mg oral tapering prednisolone; rituximab, 2x 1g i.v. rituximab; RTX, 4x 750mg i.v. rituximab.

A 28-year old Caucasian male, previously in good health with no relevant medical or family history, presented with lower extremity edema persisting for several weeks. He had not been taking any medications other than bumetanide recently prescribed by his primary care physician. Physical examination revealed hypertension and pitting edema of the legs and was otherwise unremarkable. His estimated glomerular filtration rate (Chronic Kidney Disease – Epidemiology Collaboration) was 72 ml/min per 1.73 m^2^. Further examination revealed hematuria and excessive proteinuria of 19 g/d (Figure 1, top panel).

Laboratory tests for autoimmune markers, including antinuclear antibodies, antineutrophil cytoplasmic antibodies, and antiglomerular basement membrane antibodies were negative, as were anti-streptolysin O and anti-DNase B. Complement studies revealed undetectable *in vitro* AP hemolytic activity (0%), decreased classical pathway activity (7%) and strongly reduced C3 levels (0.1 g/l), with elevated C3d (3.7%) (Figure 1, middle and bottom panel, and Table 1). A kidney biopsy was performed, which revealed mesangiocapillary and endocapillary proliferative glomerulonephritis with dominant glomerular C3 deposition. Electron microscopy showed band-like electron-dense material within the glomerular and tubular basement membranes and Bowman’s capsule, consistent with a diagnosis of dense deposit disease. Subsequent analyses demonstrated C3NeF and C5NeF positivity. Genetic testing showed no pathogenic changes in C3G-related genes. Complement factor 4 NEF, factor B, and factor H autoantibodies were absent, and there was no evidence of a monoclonal gammopathy.

**Table 1:** Overview of baseline laboratory evaluations

Parameters	Result	Reference values
Serum		
ESR	42 mm/h	<15 mm/h
Hemoglobin	7.6 mmol/l	7.5–11 mmol/l
Creatinine	121 μmol/l	<64 μmol/l
eGFR	72 ml/min per 1.73 m^2^	>90 ml/min per 1.73 m^2^
ANA	Negative	Negative
ANCA	Negative	Negative
Anti-GBM	Negative	Negative
Anti-streptolysin O	Negative	Negative
Anti-DNAseB	Negative	Negative
C3	0.1 g/l	0.9–2 g/l
C3d	3.7%	0.5–3.1%
C4	352 mg/l	95–415 mg/l
Classical pathway	7%	>74%
Alternative pathway	0%	>39%
C3NeF	83%	<20%
C4NeF	18%	<20%
C5NeF	>100%	<20%
Anti-factor H	52 AU	<200 AU
Anti-factor B	59 AU	<200 AU
M-protein	Negative	Negative
Urine		
Proteinuria	19 g/d	<0.15 g/d
Erythrocytes	203/μl	<18/μl
Leukocytes	87/μl	<10/μl

ANA, antinuclear antibodies; ANCA, antineutrophil cytoplasmic antibodies; C3, complement factor 3; C3NeF, C3 nephritic factor; C4, complement factor 4; C4NeF, C4 nephritic factor; C5NeF, complement factor 5 nephritic factor; eGFR, estimated glomerular filtration rate; ESR, erythrocyte sedimentation rate; GBM, glomerular basement membrane; M-protein, monoclonal protein.

## Results

Because of rapid deterioration of kidney function, the patient was administered 80 mg of prednisolone, resulting in a partial recovery of estimated glomerular filtration rate. Treatment was then intensified with 4 rituximab infusions of 750 mg at weekly intervals and 7 sessions of plasmapheresis. Given the persistence of proteinuria and hematuria, experimental treatments were explored in 2 clinical trials involving the complement inhibitory agents avacopan and danicopan, despite which a repeat kidney biopsy revealed persistent C3 deposition and ongoing inflammation, with progressive damage.S5,S6 In addition, AP activity remained 0% and classical pathway was still reduced at 28%. In the meantime, the patient’s condition deteriorated further, leading to end-stage kidney disease (estimated glomerular filtration rate of 11 ml/min per 1.73 m^2^) with proteinuria of about 7 g/d, prompting preparations for renal replacement therapy 3.5 years after the diagnosis.

In an effort to optimize the patient’s eligibility for transplantation considering the risk of disease recurrence, we decided to attempt antibody eradication with a B-cell targeting treatment regimen. Methylprednisolone pulses were administered at 1 g/d for 3 consecutive days. Simultaneously, he started subcutaneous belimumab of 200 mg at weekly intervals, and two 1g infusions of rituximab 4 and 6 weeks thereafter, resulting in complete peripheral B-cell depletion (<1 cells x 10^6^/l).S7 No oral glucocorticoids were administered as part of this regimen. Within 8 weeks, proteinuria markedly decreased to 1.7 g/d, and subsequently declined further to less than 0.3 g/d, with no sign of hematuria. Native kidney estimated glomerular filtration rate improved to 20 ml/min per 1.73 m^2^ and remained stable with belimumab monotherapy to this day. B cells remained reduced (about 6 cells x 10^6^/l). No adverse effects were observed throughout this period.

Importantly, biomarkers of AP activation improved noticeably, although not returning to normal. While ongoing AP activation remained, evidenced by reduced *in vitro* AP activity, increased C3d, and unchanged NeFs, the patient’s kidney function improved and proteinuria disappeared. Intriguingly, after 2 years of B-cell targeted therapy, C3NeF and C5NeF significantly declined, upon which AP activity nearly normalized (36%) and classical pathway activity completely normalized (95%).

## Discussion

With this remarkable case, we demonstrate that a synergistic B-cell targeting strategy with rituximab and long-term belimumab was highly effective in our patient with C3G, and, after a prolonged period of treatment, circulating levels of NeFs decreased.

NeFs have been postulated as either pathogenic agents, or merely as an epiphenomenon resulting from and possibly exacerbating the disease mechanism. Clearly, there is a dissociation between clinical and immunologic response to treatment, with an early clinical response and only much later a response in circulating NeFs. Nevertheless, considering their correlation with other complement biomarkers in our patient, coupled with the previous finding linking the degree of C3 convertase-stabilizing ability with disease progression, it can be concluded that eliminating these antibodies may provide a treatment advantage.^4^^,^^9^ Alternatively, the measurement of circulating NeFs may be an underestimation of the clinically relevant local NeF concentration, for example, in the glomeruli.

A complete clinical response was achieved with a combination of rituximab and belimumab, all without the need for maintenance steroids, even in the face of previous resistance to intensive immunosuppressive treatments, including rituximab. These previous treatments may have impeded complement pathway effector functions or complement factor 5a-mediated glomerular damage, temporarily altering the disease course; and this is illustrated by brief reductions in proteinuria, whereas *in vitro* measures of complement activity remained unaffected, and kidney function continued to deteriorate. The notable efficacy of our treatment approach might be attributed to a more profound and long-lasting B-cell reduction, or the enhanced targeting of memory B cells.S8 Finally, this case may illustrate the targeting of long-lived autoimmune plasma cells.

The concept of a multitargeted approach in autoimmune disease is gaining traction, and the combined use of belimumab and rituximab in particular is being increasingly recognized for its synergistic effects, possibly owing to an increased impact on lymphoid tissue and an improved targeting of memory B cells.S7,S8 Evidence from phase 2 and phase 3 clinical trials has demonstrated their effectiveness in systemic lupus erythematosus, including lupus nephritis.S9,S10 Moreover, this combination is showing promise by yielding favorable outcomes for other autoimmune disorders, such as Sjögrens syndrome and immune thrombopenia, and is currently being studied for a broader range of indications, including membranous nephropathy, antineutrophil cytoplasmic antibody-associated vasculitis and systemic sclerosis, highlighting its potential as a versatile treatment option in the field of autoimmunity.S11,S12

## Conclusions

With this case, we report the favorable clinical and immunologic outcome of a patient with C3G associated with autoantibodies, with a synergistic B-cell targeted approach. Despite the partial dissociation between clinical and immunologic response, our findings substantiate the role of NeFs in affecting the AP. Although this case may represent an exceptional scenario and its findings may not be universally applicable, the response and data supporting the response suggest a true treatment effect and warrants in-depth studies of the pathophysiology and well-designed clinical trials to ultimately unveil prognostic factors to guide treatment decisions. In the context of C3G associated with C3NeF and/or C5NeF, the potential of combined B-cell targeted therapy should be further evaluated (Table 2).

**Table 2:** Teaching points

We postulate that in cases of C3G associated with autoantibodies, profound B-cell targeting may be effective, even if complement inhibition fails.
We argue that the synergistic and long-term B-cell treatment may effectively target pathogenic long-lived plasma cells.
Clinical remission can be achieved and may not necessarily align with a complete immunologic response. Nonetheless, removal of autoantibodies may offer a treatment advantage and should be pursued.

C3G, complement factor 3 glomerulopathy.

## Disclosure

YKOT has received research support and consulting fees from GlaxoSmithKline. All the other authors declared no competing interests.

## Patient Consent

The authors declare that they have obtained consent from the patient discussed in the report.

## Supplementary Material

Supplementary File (PDF)

**Supplementary References.**
