# Supplementary material for: Clinical Remission and Reduction of Circulating Nephritic Factors by Combining Rituximab With Belimumab in a Case of Complement Factor 3 Glomerulopathy
Source: Kidney Int Rep. 2024 Feb 24;9(6):1919–22. doi: 10.1016/j.ekir.2024.02.1402 (PMC11184254; doi:10.1016/j.ekir.2024.02.1402)
Supplement: Supplementary File (PDF) [file mmc1.pdf]

Supplementary material

Supplementary references

S1. Medjeral-Thomas, N.R., et al., *C3 glomerulopathy: clinicopathologic features and predictors of outcome.* Clin J Am Soc Nephrol, 2014. **9**(1): p. 46-53.

S2. Rudnicki, M., *Rituximab for Treatment of Membranoproliferative Glomerulonephritis and C3 Glomerulopathies.* Biomed Res Int, 2017. **2017**: p. 2180508.

S3. Braun, M.C., et al., *Recurrence of membranoproliferative glomerulonephritis type II in renal allografts: The North American Pediatric Renal Transplant Cooperative Study experience.* J Am Soc Nephrol, 2005. **16**(7): p. 2225-33.

S4. Lu, D.F., et al., *Clinical features and outcomes of 98 children and adults with dense deposit disease.* Pediatr Nephrol, 2012. **27**(5): p. 773-81.

S5. Bomback, A.S., et al. *Effect of Avacopan, a Selective C5a Receptor Inhibitor, on C3G Histologic Index of Disease Chronicity*. 2021; Available from: https://www.asn-online.org/education/kidneyweek/2021/program-abstract.aspx?controlId=3639829.

S6. Nester, C., et al., *Clinical Outcomes of Patients with C3G or IC-MPGN Treated with the Factor D Inhibitor Danicopan: Final Results from Two Phase 2 Studies.* Am J Nephrol, 2022. **53**(10): p. 687-700.

S7. van Schaik, M., et al., *Efficacy of belimumab combined with rituximab in severe systemic lupus erythematosus: study protocol for the phase 3, multicenter, randomized, open-label Synbiose 2 trial.* Trials, 2022. **23**(1): p. 939.

S8. Arends, E.J., et al., *POS0680 Belimumab add-on therapy mobilises memory B cells into the circulation of patients with SLE.* Ann Rheum Dis, 2021. **80**: p. 585-585.

S9. Atisha-Fregoso, Y., et al., *Phase II Randomized Trial of Rituximab Plus Cyclophosphamide Followed by Belimumab for the Treatment of Lupus Nephritis.* Arthritis Rheumatol, 2021. **73**(1): p. 121-131.

S10. Shipa, M., et al., *Effectiveness of Belimumab After Rituximab in Systemic Lupus Erythematosus : A Randomized Controlled Trial.* Ann Intern Med, 2021.

S11. Mariette, X., et al., *A randomized, phase II study of sequential belimumab and rituximab in primary Sjogren's syndrome.* JCI Insight, 2022. **7**(23).

S12. Mahevas, M., et al., *Efficacy, safety and immunological profile of combining rituximab with belimumab for adults with persistent or chronic immune thrombocytopenia: results from a prospective phase 2b trial.* Haematologica, 2021. **106**(9): p. 2449-2457.
